# Consistency Analysis of Programmed Death-Ligand 1 Expression between Primary and Metastatic Non-Small Cell Lung Cancer: A Retrospective Study

**DOI:** 10.7150/jca.34793

**Published:** 2020-01-01

**Authors:** Luqiao Luo, Xinlan Luo, Wendan Chen, Chunyan Liang, Su Yao, Weihong Huang, Chao Liu, Yan Ge, Xingtao Lin, Zhi Li

**Keywords:** Programmed death-ligand 1 (PD-L1), non-small cell lung cancer (NSCLC), primary cancer, metastatic cancer.

## Abstract

**Background:** Programmed death-ligand 1 (PD-L1) expression in non-small cell lung cancer (NSCLC) is considered as a predictive biomarker of anti-PD-1/PD-L1 cancer therapies. However, the correlation of PD-L1 expression status between the primary and paired metastatic NSCLC is still not clear. The current study aims to address this specific issue.

**Materials and Methods:** The PD-L1 expression of the primary and paired metastatic lesions from 110 patients with NSCLC was retrospectively evaluated by immunohistochemical assay using Anti-PD-L1 antibody, Clone 22C3. The results were assessed by the Tumor Proportion Score (TPS) using cutoff values of <1%, 1%-49% and ≥50%. Meanwhile, the Cohen's kappa coefficient (*k*) of agreement was calculated.

**Results:** An overall concordance rate of the PD-L1 expression between the primary and metastatic lesions was 61% (63/103) (*k* = 0.39, and* P* < 0.001). If using TPS considering 1% and 50% as a threshold, the inconsistent rate was 28/103 (27.2%) paired specimens (*k* = 0.46, and* P* < 0.001) and 14/103 (13.6%) paired specimens (*k* = 0.53, and* P* < 0.001), respectively. Moreover, the concordance of the PD-L1 expression between primary and metastatic tumor was also analyzed according to the clinical stages within the untreated group of patients. We observed that for patients with stage I-III NSCLC, the concordance rate of the PD-L1 expression between primary and metastatic lesions was 81.3% and 100% when using 1% and 50% as threshold, respectively. While in stage IV patients, the concordance rate of the PD-L1 expression between the primary and metastatic lesions drops to 71.4% and 85.7%, respectively.

**Conclusion:** The PD-L1 expression was dynamic as tumor developed, which was valuable in selecting the proper type of sample for accurately evaluating the prognosis of using pembrolizumab as first or second line treatment.

## Introduction

Cancer is not just a gathering of a larger number of malignant cells but also a complex “barbarous” organs, to which many other cells are recruited and can be corrupted by the transformed cells. Interactions between malignant and non-transformed cells create the tumor microenvironment (TME). The non-malignant cells of the TME have a dynamic and often tumor-promoting function at all stages of carcinogenesis 1. Apart from malignant cells, the TME contains cells of the immune system, the tumor vasculature and lymphatics, as well as fibroblasts, pericytes and sometimes adipocytes. These cells are frequently distinguished by cell-type-specific markers, which are often cell surface molecules 2. Bidirectional interaction occurs between tumor cells and these resident cells types within TME, as tumor cells can secrete growth factors and cytokines that attract and modulate the behavior of both stromal cells and immune cells 3. In fact, tumors often have the means to evade detection and destruction by immune cells at almost every conceivable immune mechanistic level by recruiting immunosuppressive cells, producing immunosuppressive cytokines, developing defects in tumor antigen presentation to T-cells or by expressing negative costimulatory molecules also called T-cell checkpoint regulators, such as the cytotoxic T-lymphocyte-associated antigen-4 (CTLA-4), programmed cell death-1 (PD-1) and programmed cell death ligand-1 (PD-L1) 4-5. These immune checkpoint restoration through the use of monoclonal antibodies directed has profoundly increased the therapeutic index of anti-PD-1/PD-L1 therapies in many cancer types with particularly impressive responses observed in a subset of patients with non-small cell lung cancer (NSCLC) 6-10. Thereinto, the expression of PD-L1 has been reported in a number of human malignancies including NSCLC, in which tumor cells was assessed by immunohistochemical staining of PD-L1. In patients with metastatic NSCLC and no prior systemic therapy, pembrolizumab was approved by Food and Drug Administration (FDA) as a first line treatment for patients with PD-L1expression ≥50% based on the results of the KEYNOTE-024 (NCT02142738) study, and as a second line treatment for patients with NSCLC expressing PD-L1 in≥1% of neoplastic cells 11. Based on these data, pembrolizumab was approved in conjunction with a companion diagnostic test, the PD-L1 IHC 22C3 pharmDX assay (DAKO, Carpinteria, CA) for use on the DAKO Autostainer Link 48 (ASL48) platform 12-13. Therefore, the immunohistochemical evaluation of PD-L1 expression on the tumor specimens of NSCLC is with significant clinical diagnostic and prognostic value.

Studies to date suggest the expression of PD-L1 in tumor cells is induced by various mechanisms, not limited, but at least including oncogenic signaling and cytokines secreted from tumor-infiltrating immune cells and is closely related to the effectiveness of current PD-1/PD-L1 immunotherapy developed to date. As PD-L1 expression is largely modulated by local TME factors, it is important to have a better understanding on its expression correlation between the primary and paired metastatic NSCLCs. Earlier studies in a relatively smaller size clinical sample have already indicated a discrepancy in PD-L1 expression between primary and metastatic tumor lesions 14-15. In order to further elucidate this issue, we conducted the current study which evaluates the consistency of PD-L1 expression between the primary and metastatic NSCLCs in a relatively large clinical sample size using the well-validated 22C3 clone. At the same time, a few cases of local relapses have also been analyzed. Therefore, this study may help to establish the future guidelines to accurately evaluate the PD-L1 expression in patients, which may further improve the current treatment strategy for NSCLC.

## Materials and Methods

### Patient selection

110 patients who underwent surgical resection or aspiration biopsy of primary and metastatic tumors at the departments of pulmonary medicine and pulmonary surgery between May 2010 and November 2018 were enrolled in this study. All of the included patients met the following inclusion criteria: (1) the primary tumor histologically confirmed as non-small cell lung cancer; (2) pathologically confirmed with distant metastasis or local recurrence; (3) with or without anti-cancer therapy when first diagnosed as NSCLC. The patient demographics, tumor characteristics, adjuvant chemotherapy and follow-up data were collected from the electronic medical record system. The study was approved by the Institutional Research Ethics Committee of Guangdong Provincial People's Hospital. The clinical and pathological features of the cohort of patients are illustrated in **Table 1**.

Among the 110 patients, 103 of them had metastases (7 to regional lymph nodes and 96 to distant sites) and 7 of them had local recurrences. The sites of distant metastases included: homolateral/contralateral supraclavicular lymph node (30), pleura (22), brain (21), renicapsule (7), bone (including centum) (11), liver (1), diaphragm (1), pericardium (1), omentum majus (1) and perivascular phrenic nerve (1). Morphologic classification and tumor-node-metastasis (TNM) staging were assigned according to the current World Health Organization (WHO) and the 7^th^ American Joint Committee on Cancer (AJCC) 16.

### Samples

All tumor samples were fixed in 10% neutral buffered formalin for 8-24 hours (8-12 hours for the aspiration biopsy specimens and 12-24 hours for the surgical resection specimens). And then, specimens were blocked into a thickness of 3 or 4 mm, fixed in formalin and dehydrated and cleared in a series of alcohols and xylene, followed by infiltration with melted paraffin. Paraffin embedment was performed. Paraffin sections were cut at 4 µm and heat attached to Superfrost Plus slides in 65˚C oven for 1 hour.

### PD-L1 immunohistochemistry

PD-L1 immunohistochemistry (IHC) using mouse monoclonal Anti-PD-L1, Clone 22C3 based on EnVision^TM^ FLEX visualization system (ASL 48) was performed as it is currently the recommended assay for the detection of PD-L1 expression 17. A mouse monoclonal antibody of isotype IgG1 (Ref X0931, clone DAK-GO1, lot 20047017) was used for negative control. Deparaffinization, rehydration and antigen retrieval were performed on PT Link (Dako PT100) using the EnVision^TM^FLEX Target Retrieval Solution Low pH (pH 6.1) (Ref K8005, lot 20052704; Dako, Inc.) for 40 minutes at 97˚C. The rest of IHC assay steps were performed on ASL 48 according to the manufacturer's instructions.

### Scoring of PD-L1 expression

The PD-L1 expression of the specimen was assessed by the Tumor Proportion Score (TPS). The TPS is the percentage of viable tumor cells showing partial or complete membrane staining relative to all viable tumor cells present in the sample (positive and negative), which requests at least 100 viable tumor cells in the specimen 17:


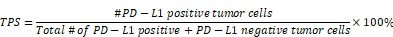


Especially, score only viable tumor cells. The following cells were excluded from scoring: infiltrating immune cell, normal cells, necrotic cells, and debris. The results were scored on a three points scale (1, 2, and 3) and thus interpreted as: no PD-L1 expression (TPS < 1%) was scored a 1, low PD-L1 expression (TPS 1%-49%) was scored a 2 or high PD-L1 expression (TPS ≥ 50%) was scored a 3 according to the previous study.

H scores, which show the change value of PD-L1 expression between primary and metastatic tumors, were calculated. H scores have a value ranging from -2 to 2 (-2, -1, 0, 1, 2) and are defined as:

*PD-L1 expression score of metastatic tumors - PD-L1 expression score of primary tumors = H scores*

### Statistical analyses

The concordance rate of PD-L1 expression between primary and metastatic tumors as well as different stages NSCLCs was compared and analyzed. At the same time, the Cohen's kappa coefficient of agreement was calculated, which was used to classify the level of concordance as: poor (<0.00), slight (0.00-0.20), fair (0.21-0.40), moderate (0.41-0.60), substantial (0.61-0.80), almost perfect (0.81-1.00) 18. The Wilcoxon signed-rank test was used for PD-L1 expression between primary and metastatic tumors comparisons, and the Mann-Whiney U test was used for comparing the PD-L1 expression between primary and metastatic tumors from two independent samples. All of the statistical analyses were performed using IBM SPSS Statistics 24.0 software. Two-sided *P* values < 0.05 were considered statistically significant.

## Results

### PD-L1 expression of the primary and paired metastatic lesions in untreated NSCLC patients

**Figure 2:**
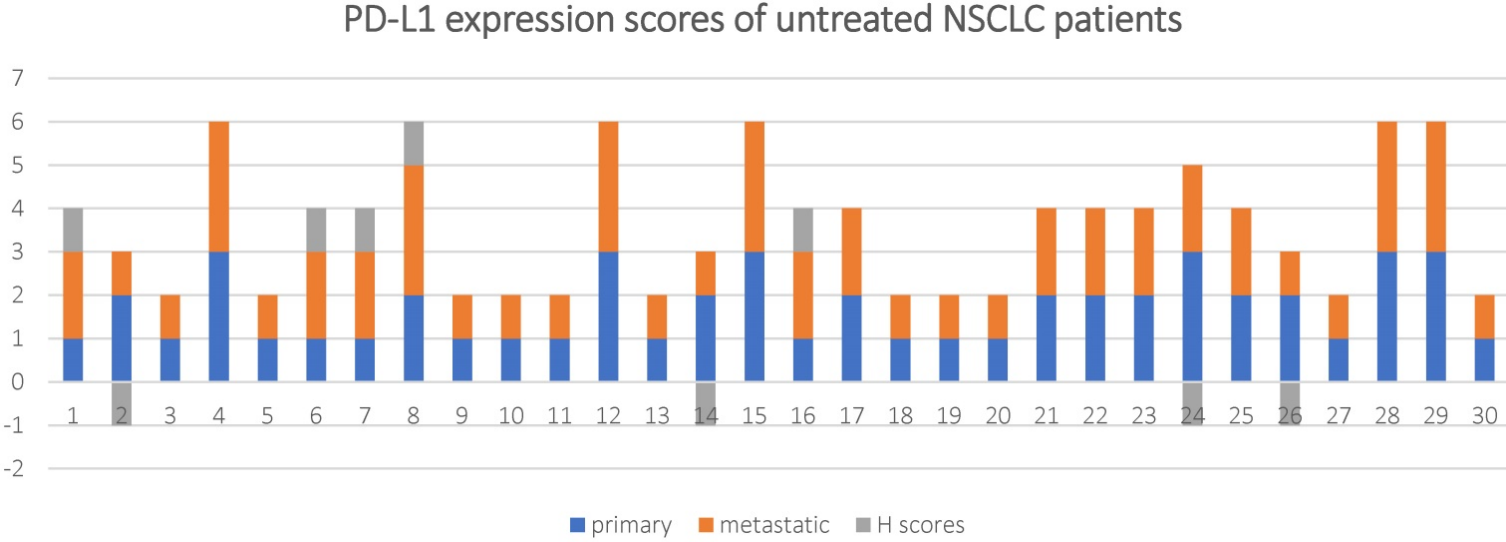
PD-L1 expression scores and H scores for in 30 untreated NSCLC patients.

**Figure 3:**
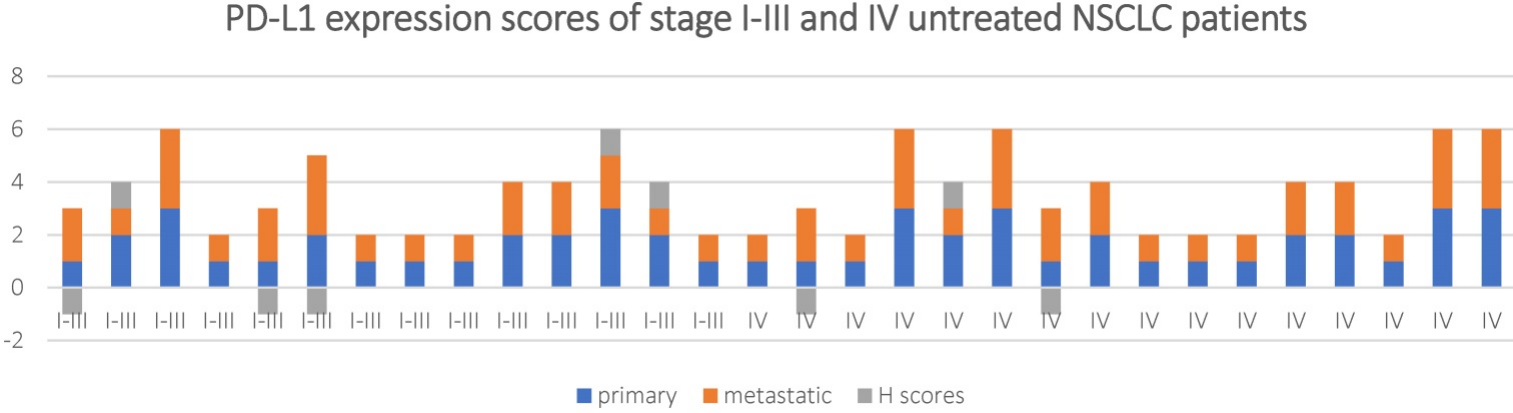
PD-L1 expression scores and H scores for in stage I-III and IV untreated NSCLC patients.

The patients in the untreated group originally underwent surgical resection or aspiration biopsy without prior chemotherapy or radiotherapy. The PD-L1 expression status and scores of their primary and metastatic lesions is shown in **Table 2, Figure 2 and Figure 3.**

The overall concordance rate between the primary and metastatic lesions was 70% (21/30) with a Cohen's kappa coefficient of 0.46 and* P* < 0.001. If 1% TPS was used as a threshold, 8/30 (26.7%) of the metastatic lesions had inconsistent PD-L1 expression compared to the primary tumor tissue (*k* = 0.54, and* P* < 0.001), among which, 7 (23.4%) cases had reduced PD-L1 expression and 1 (3.3%) case had increased PD-L1 expression in the metastasis lesions.

If 50% TPS was used as a threshold, 2/30 (6.6%)of the metastatic lesions had inconsistent PD-L1 expression compared to the primary tumor tissue (*k* = 0.78, and* P* < 0.001), among which, 1 (3.3%)cases had reduced PD-L1 expression and 1 (3.3%) case had increased PD-L1 expression in the metastasis lesions.

A Wilcoxon-signed ranks test was conducted to determine if there were differences in PD-L1 expression scores between primary and metastatic tumor in untreated NSCLC patients. The results showed that Z-value (*Z*) = -0.333, P-value (*P*) = 0.739.

The concordance of PD-L1 expression between the primary and metastatic tumor tissue for the untreated patients was analyzed based according to the clinical staging (**Table 2a and 2b**). The concordance rate of PD-L1 expression between the primary and metastatic samples for patients staging I-III was 81.3% when 1% was considered as the threshold, while it reached 100% when 50% was as the cut-off value (*k* = 0.62 and 1, and* P* < 0.001 and >0.05respectively). For stage IV patients, this number dropped to 71.4%(*k* = 0.43, and* P* < 0.001) and 85.7% (*k* = 0.42, and* P* < 0.001), respectively.

A Mann-Whitney U test was used to determine if there were differences in H scores between stage I-III and stage IV in untreated NSCLC patients. The results showed the stage I-III (mean rank = 15.11) and the stage IV (mean rank = 15.84), U-value (*U*) = 106.5,* Z* = -0.283, *P* = 0.777.

It is worth noting that, all the discordant cases were the ones with distant metastases, include: pleura (3), brain (1), renicapsule (2), homolateral/contralateral supraclavicular lymph node (3).

### PD-L1 expression of the primary and paired metastatic lesions in NSCLC patients with prior conventional treatment

**Figure 4:**
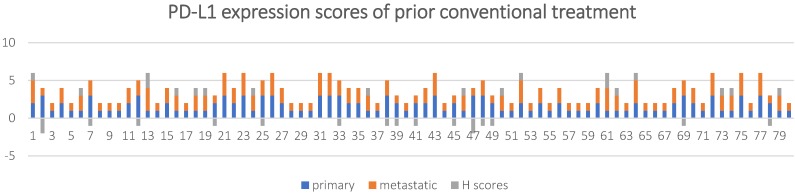
PD-L1 expression scores and H scores for in 80 NSCLC patients with prior conventional treatment.

The patients in the treated group were diagnosed as NSCLC after surgical resection or aspiration biopsy, followed by either targeted drugs treatment, chemotherapy or radiation therapy. PD-L1 expression status and scores in primary and metastatic lesions of this group of patients is shown in **Table 3 and Figure 4**.

The overall concordance rate was 60% (48/80) with a Cohen's kappa coefficient of 0.37. If 1% TPS was used as a threshold, 21/80 (26.3%) of the metastatic lesions had inconsistent PD-L1 expression compared to the primary tumor tissue (*k* = 0.45, and* P* < 0.001), among which, 18 (22.5%) cases had reduced PD-L1 expression and 3 (3.8%) case had increased PD-L1 expression in the metastasis lesions.

The results of the Wilcoxon-signed ranks test showed *Z* = -0.296 and *P* = 0.767.

A Mann-Whitney U test was run to determine if there were differences in H scores between untreated and conventional treatment in NSCLC patients. The results showed untreated patients (mean rank = 55.68) and conventional treatment in NSCLC patients (mean rank = 55.43), *U* = 1194.5, *Z* = -0.043, *P* = 0.966.

### PD-L1 expression of the primary tumors and paired distant metastatic lesions in NSCLC patients

The PD-L1 expression status in primary and distant metastatic NSCLCs with or without prior clinical treatment is shown in **Table 4**.

The overall concordance rate was 61% (63/103) with a Cohen's kappa coefficient of 0.39, and* P* < 0.001. If 1% TPS was used as a threshold, 28/103 (27.2%) of the metastatic lesions had inconsistent PD-L1 expression compared to the primary tumor tissue (*k* = 0.46, and* P* < 0.001), among which, 24 (23.3%) cases had reduced PD-L1 expression and 4 (3.9%) case had increased PD-L1 expression in the metastasis lesions.

If 50% TPS is used as a threshold, 14/103 (13.6%) of the metastatic lesions had inconsistent PD-L1 expression compared to the primary tumor tissue (*k* = 0.53, and* P* < 0.001), among which, 10 (9.7%) cases had reduced PD-L1 expression and 4 (3.9%) case had increased PD-L1 expression in the metastasis lesions.

The results of the Wilcoxon-signed ranks test showed *Z* = -0.274, *P* = 0.784.

### PD-L1 expression of the primary tumors and local recurrences in NSCLC patients

The PD-L1 expression status of the primary and local recurring NSCLCs is shown in **Table 5.**

Our results showed that, at 1% TPS cutoff, the discrepancy in PD-L1 expression was seen in 1/7(14.3%) paired metastatic sample, in which increased PD-L1 expression was observed in local recurring lesions. Due to the limited size of the current study sample, the concordance rate cannot be evaluated using 50% TPS as threshold.

The results of the Wilcoxon-signed ranks test showed *Z* = -0.1, *P* = 0.317.

A Mann-Whitney U test was run to determine if there were differences in H scores between distant metastatic lesions and local recurrences in NSCLC patients. The results showed distant metastatic lesions in patients (mean rank = 55.17) and local recurrences in NSCLC patients (mean rank = 60.36), *U* = 326.5, *Z* = -0.483, *P* = 0.629.

## Discussion

PD-L1 protein expression status evaluated by IHC assay is currently the mainstream predictive biomarker for the prognosis PD-1/PD-L1 immunotherapy. The PD-L1 IHC 22C3 pharmDx kit is an FDA approved platform to evaluate patients with metastatic NSCLC for possible pembrolizumab intervention. For NSCLC patients with a ≥ 50% TPS of PD-L1 expression, pembrolizumab can be effectively used as first-line treatment and as a second line therapy for patients with 1% TPS of PD-L1 expression 19. Thus, accurate assessment of PD-L1 expression in NSCLC tissues is critical in current clinical practice.

In fact, a majority of patients with advanced NSCLC are usually evaluated for PD-L1 expression through aspiration biopsy tissues rather than surgical resection specimens, and most of the biopsy tissues are obtained from metastatic sites. Thus, a better understanding the correlation of PD-L1expression in metastatic lesions and the related primary tumors is particularly important for accurate therapeutic treatment selection.

In this study, we not only compared the PD-L1 expression status between the primary and metastatic NSCLCs, but also that between the untreated patients and treated patients. The overall concordance rate of PD-L1 expression status between primary tumors and metastatic lesions in treated and untreated patient groups is 70% (*k*= 0.46, and* P* < 0.001) and 60% (*k*= 0.37, and* P* < 0.001), respectively. This result is consistent with results from a previous study, in which the concordance of PD-L1 expression between primary tumor and metastatic lesions from the lymph nodes based on a cohort of 66 patients was 62%; If the 1% and 50% TPS were used as a threshold, PD-L1 expression concordance were 74% and 88% respectively 20. Notably, we obtained similar concordance rates of PD-L1 expression in primary tumors and distantly metastatic lesions at 1% and 50% cutoffs, which are 72.8% and 86.4%, respectively.

For the very first time, we also analyzed the PD-L1 expression status in primary tumors and local recurrences. The discrepancy in PD-L1 expression was seen in 1/7(14.3%) paired metastatic sample at 1% cutoff. Despite of the limited sample size, the data showed that locally recurrent tumors may display a different PD-L1 expression statue compared with the primary tumors. In addition, our study demonstrated for the first time that, for NSCLC patients at relatively early stage (I-III) without prior therapy, the concordance rate for PD-L1 expression in primary and metastatic NSCLC is higher compared with those for the stage IV patients (81.3% vs 71.4%, 100% vs 85.7% at 1% and 50% threshold, respectively).

**Figure 1:**
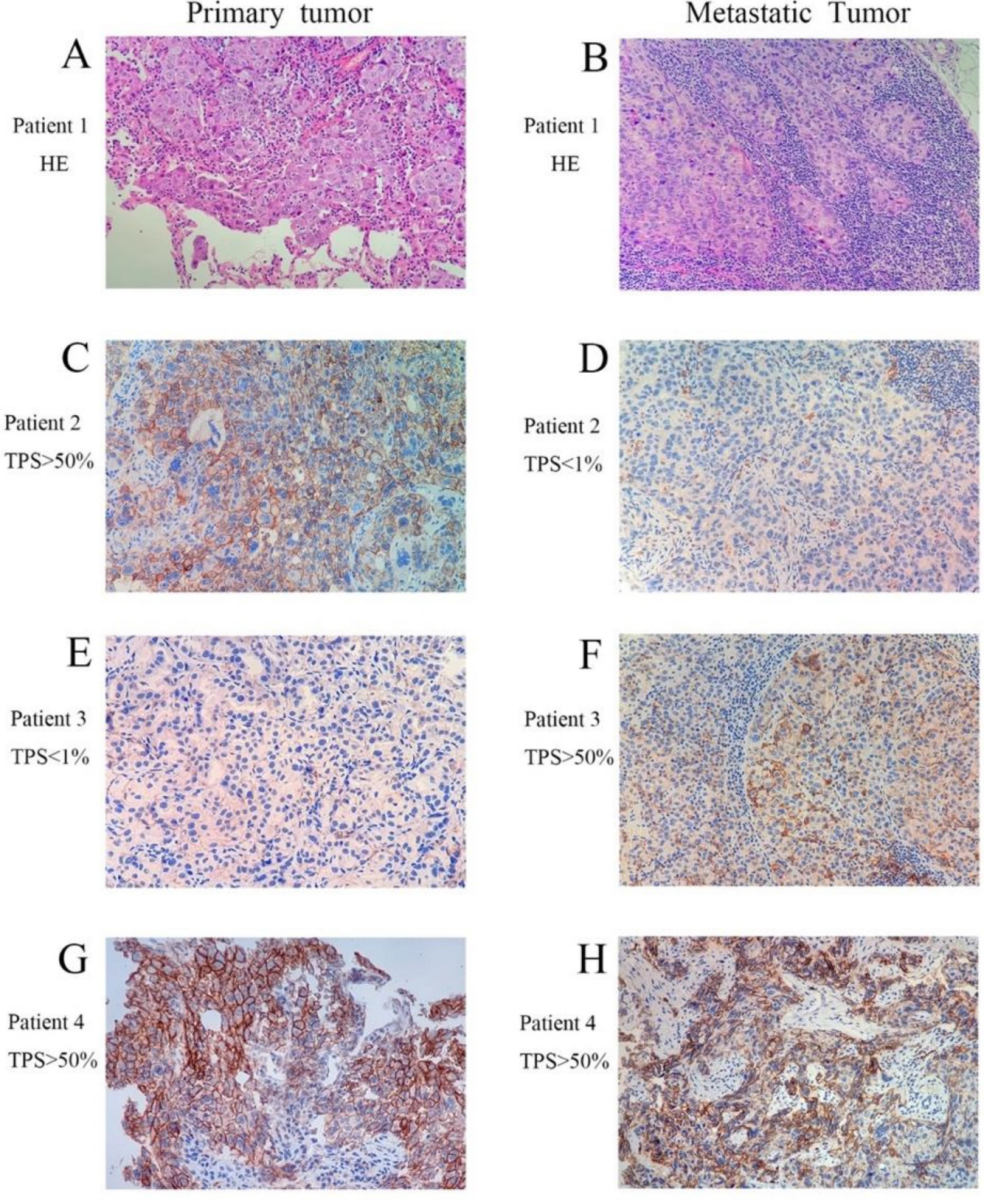
Representative images of patients in paired primary and metastatic NSCLC. (A and B, H&E staining with original magnification 400×. C-H, PD-L1 immunohistochemical staining with original magnification 400×).

Moreover, we observed that the PD-L1 expression presented a dynamic variation as tumor advanced, especially in late stage IV state (**Fig.1**). It was consistent with a recent study that significant changes of PD-L1 expression in tumor cells were observed in a part of NSCLC patients who underwent anticancer treatments. 21

The expression of PD-L1 was considered a predictive biomarker of the anti-PD-1/PD-L1 cancer therapies. Our study demonstrated that PD-L1 expression status varied in the primary and metastatic lesions or local recurrences during the clinical courses. It probably reflected that tumor cells were easily affected by the tumor microenvironment (TME) or anticancer treatment in the process of tumor development, which is consistent with earlier reports. Tumor cells could continually create a TME that was suitable for their growth and survival as the tumor increases in size 22. The intracellular signaling within TME was also dynamic during the tumor progression as a result of reciprocal signaling interactions between the tumor cells of the parenchyma and the stromal cells. When tumor cells metastasize, the circulating tumor cells were released from primary tumors and left the microenvironment created by the supportive stroma around tumor nodules. Upon landing in a distant organ, these tumor cells encountered a naive, completely different, tissue microenvironment 23. Thus, it might reflect the dynamic variations in heterotypic signaling between tumor parenchyma and stroma. In addition, as we know, cancer chemotherapy and radiotherapy did not just target malignant cells, but also on other cells in TME, which could also affect the prognosis of the treatment 24. Chemotherapy could stimulate a rapid increase in the infiltration of innate cells (e.g. chemokine and cytokine) into the damaged TME 25. Thus, the composition of the TME might be changed.

In our study, the concordance rate of PD-L1 expression between primary tumors and distantly metastatic lesions were 72.8% and 86.4% at 1% and 50% threshold respectively. While the concordance rate of PD-L1 expression between primary tumors and local recurrences was 85.7% using 1% cutoff value. Most importantly, for the first time, we observed that patients with staging I-III without previous treatment has a much better concordance rate for PD-L1 expression in primary and metastatic NSCLC than that from the staging IV group. Due to the limited sample size, the results of the Wilcoxon-signed ranks test and the Mann-Whitney U test in H scores were not statistically different. If this finding can be further verified by studies involving much larger sample size, it is acceptable to use the biopsy from metastatic site or aspiration biopsy tissues for PD-L1 expression evaluation for stage I-III NSCLC patients, but not for late stage (IV) patients. This finding might provide the guideline in selecting the proper type of sample for accurately evaluating the prognosis of using pembrolizumab as first or second line treatment at 50% and 1% threshold, respectively. Consistent with earlier studies, we also found that a lower concordance rate (60%) was detected in NSCLC patients undergoing anticancer treatments during the clinical course.

## Conclusion

In short, the PD-L1 expression was dynamic as tumor developed, which was valuable in selecting the proper type of sample for accurately evaluating the prognosis of using pembrolizumab as first or second line treatment. Future studies are required to further verify PD-L1 expression in different stages of cancer development and its relevance with therapeutic efficacy of PD-1/PD-L1 treatment. A better understanding of PD-L1 expression during tumor progression and clinical progresses will definitely advance current PD-1/PD-L1 therapies and allow the achievement of precise cancer treatment.

## Figures and Tables

**Table 1:** The clinical and pathological features of the cohort of patients

Clinical and pathological parameters	N (total = 110) (%)	
Median age (years)	60	(34-80)
Age, years		
≤60	56	(50.9)
>60	54	(49.1)
Sex		
Male	82	(74.5)
Female	28	(25.5)
Histology*		
ADC	98	(89.1)
SCC	7	(6.3)
ASC	5	(4.6)
Specimen's type		
surgical resection	54	(49.1)
aspiration biopsy	56	(50.9)
Diameter**		
≥30mm	25	(22.7)
<30mm	29	(26.4)
T stage		
pT0	2	(1.8)
pT1	22	(20.0)
pT2	32	(29.1)
pT3	19	(17.3)
pT4	35	(31.8)
N stage		
pN0	31	(28.2)
pN1	9	(8.2)
pN2	33	(30.0)
pN3	37	(33.6)
Stage (AJCC 7th)***		
I	8	(7.3)
II	8	(7.3)
III	33	(30.0)
IV	61	(55.4)
Adjuvant therapy		
NO	30	(27.3)
targeted drug/chemotherapy	79	(71.8)
radiation therapy	1	(0.9)

*: ADC: adenocarcinoma, SCC: squamous cell carcinoma, ASC: adenocarcinoma squamous cell carcinomas. **: 54 cases of surgical specimens. ***: include (a and b phase)

**Table 2:** PD-L1 expression of the primary and paired metastatic lesions in untreated NSCLC patients.

primary	metastases			
	<1%	1%-49%	≥50%	total
<1%	11	4	0	15
1%-49%	3	5	1	9
≥50%	0	1	5	6
total	14	10	6	30

**Table 2a:** PD-L1 expression of the primary and paired metastatic lesions in the untreated patients using 1% TPS as a threshold [I-III stages] (IV stages).

primary	metastases		
	<1%	≥1%	total
<1%	[6](5)	[2](2)	[8](7)
≥1%	[1](2)	[7](5)	[8](7)
total	[7](7)	[9](7)	[16](14)

**Table 2b:** PD-L1 expression of the primary and paired metastatic lesions in the untreated patients using 50% TPS as a threshold [I-III stages] (IV stages).

primary	metastases		
	<50%	≥50%	total
<50%	[12] (11)	[0] (1)	[12] (12)
≥50%	[0] (1)	[4] (1)	[4] (2)
total	[12] (12)	[4] (2)	[16] (14)

**Table 3:** PD-L1 expression of the primary and paired metastatic lesions in NSCLC patients with prior conventional treatment.

primary	metastases			
	<1%	1%-49%	≥50%	total
<1%	26	12	2	40
1%-49%	6	13	3	22
≥50%	2	7	9	18
total	34	32	14	80

**Table 4:** PD-L1 expression of the primary tumors and paired distant metastatic lesions in NSCLC patients.

primary	metastases			
	<1%	1%-49%	≥50%	total
<1%	32	15	2	49
1%-49%	9	17	4	30
≥50%	2	8	14	24
total	43	40	20	103

**Table 5:** PD-L1 expression of the primary tumors and local recurrences in NSCLC patients.

primary	metastases			
	<1%	1%-49%	≥50%	total
<1%	5	1	0	6
1%-49%	0	1	0	1
≥50%	0	0	0	0
total	5	2	0	7

## Abbreviations

AJCC: American Joint Committee on Cancer
CTLA-4: Cytotoxic T-lymphocyte-associated antigen-4
H&E: Hematoxylin-Eosin
IHC: immunohistochemistry
NSCLC: non-small cell lung cancer
PD-1: Programmed cell death-1
PD-L1: Programmed death-ligand 1
TPS: Tumor Proportion Score
TME: the tumor microenvironment
*k*: Cohen's kappa coefficient
